# A quantitative exploration of gastrointestinal bleeding in intensive care unit patients

**DOI:** 10.1371/journal.pone.0212040

**Published:** 2019-02-22

**Authors:** Patrick C. Eschenfeldt, Chin Hur

**Affiliations:** 1 Institute for Technology Assessment, Massachusetts General Hospital, Boston, MA, United States of America; 2 Gastrointestinal Unit, Massachusetts General Hospital, Boston, MA, United States of America; 3 Harvard Medical School, Boston, MA, United States of America; 4 Department of Medicine, Columbia University Medical Center, New York, NY, United States of America; University of Notre Dame Australia, AUSTRALIA

## Abstract

**Background:**

Quantitative assessments of the severity of bleeding in patients with bleeds within the gastrointestinal tract (GIB) are generally limited to blood tests like the hematocrit. The varied and irregular nature of the data collected during such observations makes it difficult in retrospective data analysis to characterize the complete course of bleeding. We intend to quantify the rate of blood loss over the course of an ICU stay, facilitating more precise analysis of retrospective data, and to use this quantification to examine questions about the effects of GIB.

**Methods and findings:**

A population of 2,445 intensive care admissions across 2,266 patients with a diagnosis of GIB was studied. Using statistical techniques for smoothing data and accepted medical approaches for calculating blood loss, we are able to convert collections of individual laboratory readings that are difficult to understand into a simple, interpretable overview of the patient’s bleeding status over time. To demonstrate this method, we compare patients’ standard vital signs while bleeding heavily to times when they are not bleeding, finding a 3.0 ± 0.5% increase in heart rate, a 1.3 ± 0.4% decrease in systolic blood pressure and a 0.9 ± 0.5% decrease in diastolic blood pressure. After considering the effect of bleeding on standard vital signs, we demonstrate that patients with upper GIB have significantly elevated blood urea nitrogen levels while bleeding heavily, with a mean increase of 11.7 ± 7.2%, while patients with lower GIB do not, with a mean increase of 4.2 ± 6.6%.

**Conclusions:**

This study introduces a novel method of processing retrospective laboratory data to characterize the course of bleeds within the gastrointestinal tract. This method is used to examine the direct effects of bleeding on a patient and can be deployed in future studies of bleeding using retrospective data.

## Introduction

Patients with or suspected to have bleeds within the gastrointestinal tract (GIB) are monitored via blood tests such as the hematocrit, along with physical observation, and frequently confirmation via endoscopy. Qualitative assessments are generally blunt and may be subjective and inaccurate, and endoscopic confirmation may be delayed and may provide little granular information about the state of the patient over the hours or days of potential bleeding leading up to the endoscopy. As such, when considering quantitative data, laboratory values provide the most complete data to characterize the course of a GIB. Such values, however, are recorded at irregular intervals and may not be directly interpretable as measures of the severity of bleeding. It was therefore our aim to develop techniques for processing this data into a form that is more useful for GIB research.

Using a database of intensive care unit (ICU) patients with a diagnosed GI bleed, we used established statistical methods for smoothing and interpolating data to generate a continuous estimate of the hematocrit level for each patient over their entire ICU stay. We then used analytical methods developed in the anesthesiology literature to convert the hematocrit level into a continuous estimate of blood loss. This form of data facilitates simple characterization of the severity of bleeding at any moment during a patient’s admission, rather than relying on blunt rules of thumb for interpreting hematocrit readings over time.

To demonstrate the legitimacy of this technique, we studied the physiological effects of GI bleeding by comparing individual patients’ vital signs while bleeding to parallel measurements while not bleeding. We first assessed changes to standard vital signs assuming that changes in heart rate and blood pressure correlated to active bleeding or provided biological support for our predictions. We then performed an exploratory analysis of the effect of GI bleeding on blood urea nitrogen (BUN) levels. Elevated BUN has been shown to be associated with ICU admission among patients presenting with upper GI bleeds [1], but its value as a predictor of outcomes has had conflicting results [2, 3]. Using our quantified estimate of bleeding rate we can investigate the relationship between BUN and severity of bleeding. Similarly, in light of research showing creatinine level at admission is a risk factor for GIB [4] we can investigate the relationship between creatinine and GIB.

Another measure of the severity of bleeding is the classification system provided by The Advanced Trauma Life Support (ATLS) course [5], which separates hypovolemic shock into four classes. These classes represent percentages of blood lost, with classes I through IV indicating less than 15%, 15−30%, 30−40%, and greater than 40%, respectively. Assignment of patients into classes is done via heart rate, blood pressure, pulse pressure, respiratory rate, mental status, and urine output. The conditions for each class are summarized in Table 1. We also compared our estimates of blood loss to the ATLS classifications.

**Table 1:** The ATLS classification of hypovolemic shock.

	Class I	Class II	Class III	Class IV
Blood loss (%)	< 15	15−30	30−40	> 40
Heart rate	< 100	100−120	120−140	> 140
Blood pressure	Normal	Normal	Decreased	Greatly decreased
Pulse pressure	Normal or increased	Decreased	Decreased	Decreased
Respiratory rate	14−20	20−30	30−40	> 35
Mental status	Slightly anxious	Mildly anxious	Anxious, confused	Confused, lethargic
Urine output (mL/hr)	> 30	20−30	5−15	Minimal

## Methods

### Data collection and study population

The Medical Information Mart for Intensive Care (MIMIC) III database [6, 7] contains deidentified data for critical care patients at Beth Israel Deaconess Medical Center between 2001 and 2012. It includes a variety of information for each patient, including diagnosis codes and time-stamped laboratory and vital sign measurements. Using the MIMIC III database version 1.4, admissions with a billing diagnosis for GI bleed were identified, using the ICD 9 codes 578.0, 578.1, and 578.9. A total of 2,451 admissions were identified, involving 2,272 patients. Patients under 18 years old at the time of admission (n = 6) were excluded from analysis, leaving a population of 2,445 admissions comprised of 2,266 patients.

### Overview of approach

We performed a smoothing operation on hematocrit readings to generate an estimate of the hematocrit level at all times during each admission rather than only the times when readings were actually recorded while also mitigating the noise in such readings. This continuous estimate of hematocrit level was then used to generate an estimate of blood loss rate to allow direct interpretation of the patient’s bleeding status at any time during the admission. We confirmed our approach by checking that the physiologic responses to estimated bleeding rates aligned with expected responses to bleeding, then analyzed the response of BUN and creatinine to estimated bleeding as illustrative examples of the kind of exploratory analysis made possible by this method. Finally we compared our method to a gold standard classification of bleeding in a somewhat different context to highlight the similarities and differences.

### Hematocrit smoothing

All available hematocrit readings were gathered, and a cubic smoothing spline was fit to these data points for each admission. These fits were computed using Python 3.6 [8] with the package SciPy [9]. A smoothing spline approximates a set of values by generating a piecewise polynomial function which has equal slope and curvature on either side of each transition point between polynomial sections. These transition points are called “knots”, and as more knots are included in the fit the shape is allowed to fluctuate more dramatically and thus the fit becomes less smooth. The algorithm used for fitting a cubic smoothing spline [10–12] includes one parameter, which controls the balance between accurately representing the individual data points and having a smoother resulting function. Specifically, given data points (*x*_*i*_, *y*_*i*_) to approximate, and resulting smoothing function *f*, the parameter *s* imposes the restriction

$$\begin{array}{c} \sum_{i}{\left(y_{i}-f\left(x_{i}\right)\right)}^{2}\leq s, \end{array}$$

and the number of knots is increased until this condition is satisfied. If *s* = 0 then *f* will exactly match all data points, becoming an interpolation, and if *s* = ∞ no knots will be used and *f* will be a simple cubic function. Via qualitative visual examination of the resulting fits on several admissions with various values for *s*, we selected the value *s* = 0.2*n*_*o*_, where *n*_*o*_ is the number of observed hematocrit readings for the admission.

The computed smoothing splines cover the time period between the first and last hematocrit readings of the admission. For any time between admission and discharge outside that window the hematocrit level was considered to be unknown. We also defined the hematocrit as unknown for any time where the smoothing spline estimate was either negative or above 100%.

### Blood loss estimation

Using the smoothing spline estimates of hematocrit, a continuous estimated rate of blood loss was computed for each admission. This computation is based on the work of Bourke and Smith [13], and their theoretical blood dilution equation. Let *H* be the hematocrit level, *dH* be the rate of change of the hematocrit level, *V* be the estimated blood volume, and *dL* be the rate of blood loss. Then the equation is

$$\begin{array}{c} \frac{dH}{H}=\frac{dL}{V}. \end{array}$$

This theoretical formula has been used to estimate blood loss during surgery, though often in a simplified form to aid calculations [14, 15]. To compute the blood loss from the estimated hematocrit, the differential equation can be rearranged as

$$\begin{array}{c} dL=V\frac{dH}{H}. \end{array}$$

In applying this equation, we used the smoothing spline estimate of hematocrit level for *H*, and computed the derivative of the smoothing spline estimate to use as *dH*. Blood volume was estimated for each admission using available patient information as follows: if height and weight were recorded for the admission then the Nadler [16] formula is used, if only weight is available then average blood volumes of 75 mL/kg for men and 65 mL/kg for women are used, with the average weight for the gender in the studied patient population used if no weight is recorded for a particular patient.

### Physiological and laboratory reactions

Using these continuous estimates of blood loss rate, every minute of each admission, from the admission time to discharge, was classified by whether the patient is bleeding (*dL* > 0 mL/min) or not bleeding (*dL* ≤ 0 mL/min). Those where the patient is bleeding were further categorized as follows: very light (0−0.1 mL/min), light (0.1−0.5 mL/min), heavy (0.5−5.0 mL/min), and very heavy (> 5.0 mL/min). Any minute for which there was no estimated hematocrit level because it was either outside the window of time with hematocrit readings or the estimated level was invalid was classified as “unknown”.

Time-stamped recorded measurements of heart rate, blood pressure (systolic and diastolic), oxygen saturation, temperature, and respiration rate were gathered for each admission. Measurements were aligned with the blood loss classifications described above so each could be associated with the patient’s blood loss status at that moment in time. For any cases where a single minute had multiple measurements the values were averaged together. For each patient, the average value when the patient was in each of the defined bleeding states was computed. The relative difference, in percentage terms, between each of the states and the non-bleeding state was computed for each patient. The mean of these differences across patients was computed, and a student’s t-test was performed to assess whether the mean was nonzero, with p-value of 0.05 as the threshold for significance.

An identical analysis was performed on blood urea nitrogen (BUN) and creatinine measurements, in the former case including only patients whose admitting diagnosis was delineated specifically a lower or upper GIB, with the analysis performed separately for upper and lower.

### Comparison to ATLS classification

For each admission with an estimated blood loss, we gathered all values of the components of the ATLS classification recorded during the admission. Following [17], we converted the subjective ATLS criteria into numerical cutoffs. For systolic blood pressure we treated ≥ 110 mmHG as class I, ≥ 100 mmHG as class II, < 100 mmHG as class III, and < 90 mmHG as class I. For mental status, we used the Glasgow Coma Scale [18], treating “slightly anxious” and “mildly anxious” as a GCS of 15, “anxious, confused” as a GCS of 12−14 and “confused, lethargic” as a GCS of less than 12. We computed pulse pressure as diastolic subtracted from systolic blood pressure and treated values ≥ 40 mmHG as “normal or increased” and values < 40 mmHG as “decreased”. Because urine output was not recorded consistently for all admissions it is not included in this analysis.

For every time during each admission where all five of these components were recorded, an ATLS classification was computed. First this was done strictly following the definitions given above, with a particular class being assigned if and only if the recorded values were all consistent with a given class. Because it has been shown many trauma patients do not fall into any class [17, 19] when using this method, we then assigned classes according to which class agreed with the most components. For example, if heart rate, pulse pressure, and respiratory rate all fell within the ranges allowed in class II, but blood pressure and mental status did not, the measurement would be assigned class II. For both methods of calculating ATLS classification, the median estimated rate of blood loss was computed at the time of classification.

## Results

### Blood loss estimation

**Fig 1:**
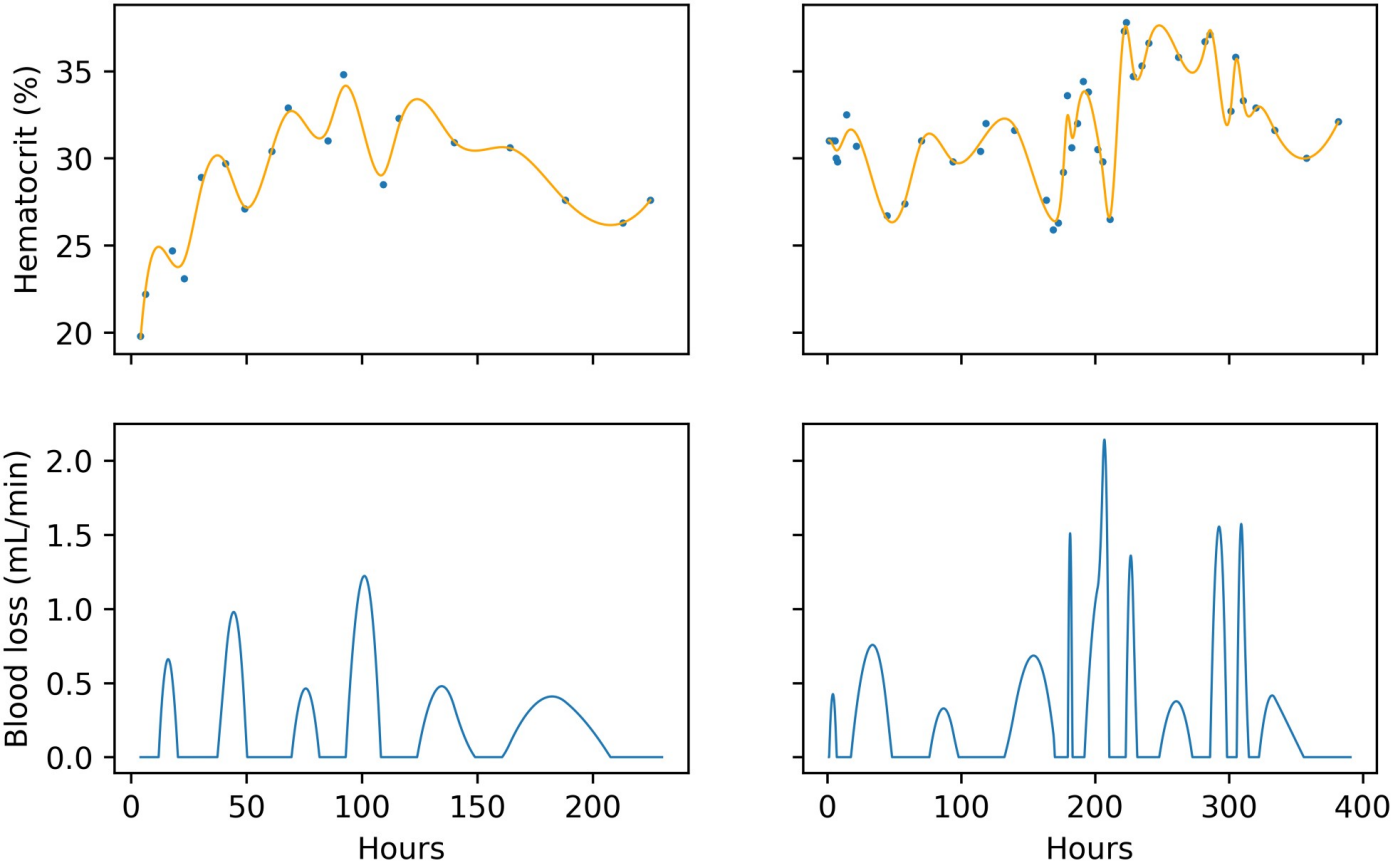
Blood loss estimation examples. Smoothed hematocrit estimates (top) and blood loss rates (bottom). Blue points represent hematocrit readings. Left and right panels are two different admissions. Negative blood loss rate is set to zero.

See Fig 1 for examples of the estimated rate of blood loss over the course of an admission.

### Physiological reactions

Heart rate was significantly higher for all categories other than very light bleeds, with a mean increase of 1.7 ± 0.4% for any bleed relative to not bleeding. The increase was 3.0 ± 0.5% for heavy bleeds and 5.0 ± 1.4% for very heavy bleeds. Full results are summarized in Table 2.

**Table 2:** Effect of bleeding on heart rate.

Bleeding	Mean Difference (%)	Admission Count	Measurements per Admission	p-value
None	Reference	2269	84.7	-
Very Light	0.0 ± 0.4	1889	20.0	0.9209
Light	0.7 ± 0.4	2062	42.7	0.0010
Heavy	3.0 ± 0.5	1881	36.3	< 0.0001
Very Heavy	5.0 ± 1.4	460	11.1	< 0.0001
Any Bleed	1.7 ± 0.4	2172	90.9	< 0.0001
Unknown	2.4 ± 1.2	760	20.3	0.0001

Both systolic and diastolic blood pressure were significantly lower for heavy bleeds, and the difference in systolic blood pressure is also significant for very heavy bleeds. The mean difference for heavy bleeds was larger for systolic blood pressure at -1.3 ± 0.4% compared to -0.9 ± 0.5% for diastolic blood pressure. Results are summarized in Table 3 for systolic blood pressure and Table 4 for diastolic blood pressure. In both cases the largest observed mean difference was that blood pressure was significantly lower for periods with unknown bleeding status, though fewer patients were in this category than all of the bleeding categories except for very heavy bleeds. Further breakdown of unknown bleeding status indicates that the effect is primarily driven by blood pressure measurements after the final hematocrit reading, but diastolic blood pressure is significantly higher before the first hematocrit reading. Neither blood pressure is significantly different than the baseline during periods of time where the estimation technique used in this paper fails to produce a valid estimate because the smoothing estimates an invalid hematocrit level. Such gaps contain blood pressure data for 64 admissions of the 2269 admissions used to define the reference group.

**Table 3:** Effect of bleeding on systolic blood pressure.

Bleeding	Mean Difference (%)	Admission Count	Measurements per Admission	p-value
None	Reference	2269	75.7	-
Very Light	0.2 ± 0.4	1876	18.9	0.2276
Light	0.2 ± 0.4	2057	38.9	0.2643
Heavy	−1.3 ± 0.4	1881	34.2	< 0.0001
Very Heavy	−2.8 ± 1.2	458	9.8	< 0.0001
Any Bleed	−0.3 ± 0.3	2173	84.1	0.0840
Unknown	−5.8 ± 1.2	754	16.6	< 0.0001

**Table 4:** Effect of bleeding on diastolic blood pressure.

Bleeding	Mean Difference (%)	Admission Count	Measurements per Admission	p-value
None	Reference	2269	75.7	-
Very Light	0.1 ± 0.5	1876	18.9	0.06892
Light	−0.3 ± 0.5	2057	38.9	0.2656
Heavy	−0.9 ± 0.5	1881	34.2	0.0009
Very Heavy	−1.0 ± 1.4	458	9.8	0.1602
Any Bleed	−0.3 ± 0.4	2173	84.0	0.0756
Unknown	−3.8 ± 1.3	753	16.7	< 0.0001

Other vital signs demonstrate similar patterns, with respiration rate significantly increased while bleeding, temperature slightly but significantly increased while bleeding, and oxygen saturation significantly increased during heavy and very heavy bleeding. Full results can be found in S1, S2 and S3 Tables, respectively.

### BUN

For admissions where the admitting diagnosis was an upper GI bleed (n = 315), the BUN was significantly higher for heavy bleeds, but not any other category of bleed. The mean increase in BUN during heavy bleeds was 11.7 ± 7.2%. Results are summarized in Table 5.

**Table 5:** Effect of bleeding on BUN in patients admitted for upper GI bleed.

Bleeding	Mean Difference (%)	Admission Count	Measurements per Admission	p-value
None	Reference	291	4.8	-
Very Light	−1.4 ± 6.5	122	2.2	0.6655
Light	1.7 ± 6.0	188	2.9	0.5729
Heavy	11.7 ± 7.2	179	2.1	0.0016
Very Heavy	3.8 ± 19.1	34	1.3	0.6941
Any Bleed	3.3 ± 4.8	261	4.6	0.1826
Unknown	−0.1 ± 20.9	7	2.0	0.9886

In patients admitted with a lower GI bleed (n = 251), BUN was significantly lower for very light bleeds, with a mean difference of -7.3 ± 5.9% relative to the baseline. Heavy and very heavy bleeds were not associated with a significant difference in BUN. Results are summarized in Table 6.

**Table 6:** Effect of bleeding on BUN in patients admitted for lower GI bleed.

Bleeding	Mean Difference (%)	Admission Count	Measurements per Admission	p-value
None	Reference	234	4.6	-
Very Light	−7.3 ± 5.9	94	2.3	0.0169
Light	−3.9 ± 4.8	155	2.6	0.1150
Heavy	4.2 ± 6.6	160	2.0	0.2132
Very Heavy	14.0 ± 24.7	22	1.3	0.2529
Any Bleed	−1.3 ± 4.5	219	4.3	0.5712
Unknown	−4.3 ± 21.9	12	1.3	0.6726

### Creatinine

Creatinine was significantly higher for any bleed, with a mean difference of 7.2 ± 5.2%. This increase even larger for heavy bleeds at 12.3 ± 7.7% and also significant for very heavy bleeds and when bleeding status was unknown, primarily after the last hematocrit reading. Full results are in Table 7.

**Table 7:** Effect of bleeding on creatinine.

Bleeding	Mean Difference (%)	Admission Count	Measurements per Admission	p-value
None	Reference	1827	5.3	-
Very Light	1.0 ± 1.7	692	2.7	0.2797
Light	4.7 ± 6.4	1060	3.8	0.1507
Heavy	12.3 ± 7.7	1094	3.2	0.0018
Very Heavy	5.3 ± 4.3	229	1.5	0.0160
Any Bleed	7.2 ± 5.2	1477	6.3	0.0066
Unknown	8.0 ± 7.0	177	1.2	0.0248

### Comparison to ATLS classification

A total of 1,240 admissions had at least one time when the five components of the ATLS classification were observed. There were a total of 52, 537 observations, and 47, 016 (89%) did not correspond to an exact ATLS class. Among the 5, 521 which could be classified, 5, 438 (98%) were class I, with 79, 2, and 2 observations in classes II, III, and IV, respectively. For class I the median [interquartile range] instantaneous estimated rate of blood loss was −0.03 [−0.43, 0.31] mL/min, for class II −0.03 [−0.39, 0.44], for class III 1.24 [0.76, 1.71], and for class IV −0.06 [−0.07, −0.05]. For those observations that could not be classified the estimated rate of blood loss was 0.03 [−0.29, 0.35].

Using the alternate method of computing ATLS classes, all observations were placed into one of the four classes. Of the 52, 537 total, 41, 793 (80%) were class I, 8, 172 (16%) were class II, 1, 452 (3%) were class III, and 1, 120 (2%) were class IV. For class I the median instantaneous estimated rate of blood loss was 0.02 [−0.21, 0.33] mL/min, for class II 0.05 [−0.29, 0.40], for class III 0.10 [−0.26, 0.48], and for class IV 0.04 [−0.36, 0.49].

## Discussion

We present a technique for quantifying the rate of blood loss during the course of a hospital stay based on a patient’s measured hematocrit levels. By combining a standard blood test for monitoring the status of bleeding patients with standard statistical techniques for smoothing data and with accepted medical approaches for calculating blood loss, we are able to convert messy collections of individual laboratory readings into a simple, interpretable overview of the patient’s state over time. This estimate allows us to retroactively analyze the effects of bleeding in an intuitive way, which we demonstrate by first examining standard vital signs. We see that while patients are bleeding their heart rate is significantly higher than when they are not bleeding, and when they are bleeding heavily their blood pressure is significantly lower, aligning with standard expectations [20]. These findings provide some limited evidence that our estimation technique accurately reflects bleeding status.

We note, however, that the observed effects of bleeding are very small on average, even though they are generally significant, at least for heavy bleeds. One potential reason for this is that some patients may have idiosyncratic reactions to bleeding [21], affecting population averages. Another likely factor is the instantaneous nature of the comparisons being performed: we classify each minute of a hospital stay by the estimated bleeding status at that exact moment, ignoring the patient’s state at times before and after. Many physiologic responses will not take effect instantaneously or return to a normal state immediately when bleeding stops, so vital signs should be expected to be different for a patient who is currently bleeding lightly but was bleeding heavily for the previous 6 hours than for a patient who just began bleeding lightly after not bleeding for hours. Future studies could use the complete estimated course of bleeding to define more complex patient states than our simple “present state” categories, which we select for ease of understanding and to avoid splitting the data into unrepresentatively small pieces. We consider the simpler comparisons a useful measure of the directional effect of bleeding, and the relative differences between those effects for different levels of bleeding, but do not consider them to be accurate measures of the quantitative effect of bleeding generally.

One area in which the existence and direction of an effect is of particular interest is the BUN. We show that BUN is significantly elevated during heavy bleeding for patients admitted with upper GI bleeds but is not significantly different for lighter bleeds. For lower GI bleeds, no severity of bleeding showed a significant increase in BUN, with lighter bleeds in fact showing a significant decrease. Prior studies had reported conflicting results regarding the validity of BUN as a predictor of active upper GI bleeding, perhaps because of the time delays between BUN reading and determination of active GI bleeding by endoscopy which was likely many hours later.

Comparisons of our approach to the ATLS gold standard classification of hypovolemic shock demonstrate that there are major differences between the two methods, with all ATLS classes demonstrating similar instantaneous estimated bleeding behavior. We believe the primary reason for this lack of correlation is that the two classification methods are intended for fundamentally different tasks, and will primarily be applied in different settings. ATLS classification is intended for trauma patients and attempts to quantify the amount of blood that has been lost by characterizing the physiological response to that loss of blood. In contrast, our estimated blood loss attempts to quantify how much blood is being lost at a particular moment in time, regardless of how much has been lost before that time. It is intended to quantify the patient’s state over an extended period of time, which likely includes periods of bleeding interspersed with periods of minimal or no bleeding. Just as alternatives to ATLS classification have been suggested [22] in the context of shock in trauma patients it is likely that alternative and improved methods exist for the estimation of instantaneous blood loss. We believe, however, that the method proposed here is more appropriate in this context than adaptations of the methods for classifying the severity of shock in trauma patients.

This study is limited by using only patients from a single site, and by focusing solely on ICU patients, who may not be representative of broader patient populations of interest. However, the primary technique introduced here to generate continuous blood loss estimates from hematocrit levels is not dependent on the patient population and may be applied to any patient or group of patients for whom sufficient hematocrit measurements are available.

A limitation of the method used in this study is that it relies only on hematocrit readings and does not account for factors other than blood loss which might affect the hematocrit, such as urinary losses, fluid replacement, and transfusions. Developing a complete and accurate physical model of the interaction between hematocrit and bleeding is implausible even if complete information about these factors were available, which it often is not. We believe that the imperfect and simplified model used in this study is sufficiently informative to be useful in contexts where a continuous characterization of bleeding is required.

We also note that this technique could be generalized into a framework of approaches to convert hematocrit readings into continuous estimates of blood loss. The smoothing spline portion of our technique can be replaced with any appropriate technique for smoothing and interpolating data, including techniques that draw on data from multiple patients to learn plausible shapes rather than relying on simple cubic splines. More complex approaches could also be better tailored to avoid the “unknown” regions resulting from the simplicity of the smoothing splines model, at the expense of harder computations and potentially more reliance on the availability of representative data to train such a method. The simpler approach taken here is easier to compute and understand, and also has the advantage of providing an analytically computable derivative to use when calculating blood loss from hematocrit, avoiding reliance on numerical derivatives or an additional layer of smoothing to generate a differentiable estimate.

While we believe the BUN correlation with upper GIB is of interest, the blood loss estimates used to generate them may be used for a wide variety of more complex analyses. One setting for which they are particularly well suited is that of personalized, real-time identification and prediction of GI bleeds. In this setting, it is valuable to have continuous estimates of bleeding so that precise times can be associated with events such as the start of bleeding or a significant increase in the rate of bleeding. By providing tools to make GI bleeding data easier to quantify and interpret, we hope to facilitate future research making it easier to understand and predict.

## Supporting information

S1 Table Effect of bleeding on respiration rate. (PDF)

S2 Table Effect of bleeding on temperature. (PDF)

S3 Table Effect of bleeding on oxygen saturation. (PDF)
