# Supplementary material for: A quantitative exploration of gastrointestinal bleeding in intensive care unit patients
Source: PLoS One. 2019 Feb 22;14(2):e0212040. doi: 10.1371/journal.pone.0212040 (PMC6386222; doi:10.1371/journal.pone.0212040)
Supplement: S1 Table — (PDF) [file pone.0212040.s001.pdf]

Supplementary Table 1: **Effect of bleeding on respiration rate.**

Bleeding	Mean Difference (%)	Admission Count	Measurements per Admission	p-value
None	Reference	2261	83.2	-
Very Light	1.1 ± 0.7	1873	19.8	0.0015
Light	1.1 ± 0.6	2046	42.1	< 0.0001
Heavy	0.7 ± 0.6	1872	35.3	0.0239
Very Heavy	0.5 ± 2.0	451	10.8	0.6278
Any Bleed	1.2 ± 0.5	2157	89.1	< 0.0001
Unknown	2.1 ± 1.6	748	19.8	0.0117
