# Supplementary material for: A quantitative exploration of gastrointestinal bleeding in intensive care unit patients
Source: PLoS One. 2019 Feb 22;14(2):e0212040. doi: 10.1371/journal.pone.0212040 (PMC6386222; doi:10.1371/journal.pone.0212040)
Supplement: S2 Table — (PDF) [file pone.0212040.s002.pdf]

Supplementary Table 2: **Effect of bleeding on temperature.**

Bleeding	Mean Difference (%)	Admission Count	Measurements per Admission	p-value
None	Reference	2234	19.6	-
Very Light	0.0 ± 0.0	1379	6.0	0.0987
Light	0.1 ± 0.0	1786	10.4	< 0.0001
Heavy	0.1 ± 0.1	1773	7.7	0.0002
Very Heavy	-0.3 ± 0.4	329	2.3	0.1855
Any Bleed	0.1 ± 0.0	2101	19.4	< 0.0001
Unknown	-0.2 ± 0.3	632	3.6	0.1202
