# Supplementary material for: A quantitative exploration of gastrointestinal bleeding in intensive care unit patients
Source: PLoS One. 2019 Feb 22;14(2):e0212040. doi: 10.1371/journal.pone.0212040 (PMC6386222; doi:10.1371/journal.pone.0212040)
Supplement: S3 Table — (PDF) [file pone.0212040.s003.pdf]

Supplementary Table 3: **Effect of bleeding on oxygen saturation.**

| Bleeding | Mean Difference (%) | Admission Count | Measurements per Admission | p-value |
|---|---|---|---|---|
| None | Reference | 2268 | 84.1 | - |
| Very Light | $-0.1 \pm 0.1$ | 1866 | 20.5 | 0.4288 |
| Light | $0.0 \pm 0.1$ | 2047 | 43.2 | 0.4063 |
| Heavy | $0.3 \pm 0.3$ | 1877 | 36.0 | 0.0451 |
| Very Heavy | $0.3 \pm 0.3$ | 462 | 10.9 | 0.0901 |
| Any Bleed | $0.2 \pm 0.3$ | 2165 | 91.2 | 0.2274 |
| Unknown | $-2.0 \pm 0.6$ | 734 | 16.7 | $< 0.0001$ |
